# Achievement motivation level in students of Shiraz University of Medical Sciences and its influential factors

**Published:** 2015-01

**Authors:** SOMAYEH KAVOUSIPOUR, ALI NOORAFSHAN, SAEEDEH POURAHMAD, ALI DEHGHANI-NAZHVANI

**Affiliations:** 1 School of Rehabilitation, Shiraz University of Medical Sciences, Shiraz, Iran;; 2 Histomorphometry & Stereology Research Center, Shiraz University of Medical Sciences, Shiraz, Iran;; 3 Biostatistics Department, Shiraz University of Medical Sciences, Shiraz, Iran;; 4 Depatment of Oral & Maxillofacial Pathology, Biomaterial Research Center, School of Dentistry, Shiraz University of Medical Sciences, Shiraz, Iran;

**Keywords:** Academic, Motivation, Education, Students, Effective

## Abstract

**Introduction**: Many studies have investigated the relationship between motivation and educational outcomes. The present study was conducted to determine whether the students’ motivation in Shiraz University of Medical Sciences (SUMS) decreases during educational years.

**Methods**: 770 students in SUMS were selected by multi-stage stratified random sampling from each field and entrance year. The first questionnaire contained 57 questions on the effect of economic, social, educational, geographical and personality factors on the students’ motivation. The second one was based on 50 incomplete sentences. The validity and reliability of these questionnaires were approved by the experts and Cronbach's Alpha coefficients (85% and 90%, respectively). In this cross-sectional study, ANOVA, t-test and Chi-square tests were applied for data analysis at the 0.05 significance level.

**Results**: Six factors with the most effect on academic motivation were "family attitudes", "getting good jobs in future", "respect for themselves", " the ability to learn", "believing their role in victory and defeat" and "the tendency toward optimism about themselves". In addition, comparing professional doctorate and basic sciences’ results revealed no significant relationship between academic motivation and educational years (F=0.819, p=0.397). But comparing field by field showed that Dentistry and Hospital Management and Medical Information (HMMI) had a significant decrease in motivation score by increase in educational years (F=3.991, p=0.015).

**Conclusion**: Achievement motivation level in SUMS students was higher than average and did not decrease during educational years. Also, the results showed that personal, social and educational related factors affected motivation level more than economic and environmental factors.

## Introduction

Motivation is a complex part of human psychology and behavior that influences how individuals choose to invest their time, how much energy they exert in any given task, how long they persist in the task, etc (1-4).

Some psychologists and theories such as Freud, Mcclelland and Mory viewed motivation as primary result of drives and needs and perceived it as intra-individual factors. They developed several biological and psychological needs for human. For example promotion is a social need and it means, overcoming barriers, achieving a high standard and competing with others (5). Human behavior arises from his/her needs and promotion is one of the basic needs of human behavior. Evidence shows that there are some differences in human needs to promotion in different individuals. Some people have high levels of need, so they try hard to overcome the problems. Some others have fear of failure and don’t strive to promote (6). Some other theories focused on contextual features that affect goal achievement, so they believe that punishment or reinforcement can reduce or enhance task engagement. More recently, motivation research has focused on social cognitive perspective; according to this view, belief about their academic ability and expectations about the outcomes of engaging in the task etc. are influenced by social contextual factors (4, 7, 8).

As mentioned, there are some intrinsic and extrinsic factors that can induce motivation. Although with each theory we try to define motivation and philosophy of motivation, the level of motivation is important for goal attainment. Pent Reach and Shraben believe that the level of motivation affects the achievement of goals and amount of affordance (9, 10).

Different studies have shown that there are many stimulators to move the students forward, i.e. conducting behavior toward specific goals, increasing affordance, increasing activity level, hard working, functional improvement, positive emotion (hope and pride), self satisfaction, enjoyment from learning activity, family condition, gender of the students, number of family members, parents’ job, and economical status (11-17).

Today, lack of interest in learning and motivation in students is important in difficulties for educational system that offer a low level of quality for learning. The amount of the students' motivation even interferes with social development (18). Mckeachie indicated that 83% of he graduated students with a high level of motivation require a kind of job that needs difficult planning, making decisions and can lead to high probability of unbelievable success (19). These students believe that they can make their future by themselves. Since motivation has an essential role in the students’ learning and their educational improvement, it is necessary that educational systems find some ways and solutions to develop it and find factors that affect its level, such as social, psychological and familial factors (20) .

The culture of students can affect achievement motivation, so research has found that European students like to be in the school because of art and sports activity; on the other hand, western students like to be in school because of its opportunity for social positions and improvements. Areepattamannil found that intrinsic and extrinsic motivation both could affect mathematic achievement among Indian immigrant adolescents in Canada, but there was no significant relationship between motivation and achievement among Indian adolescents in India, so this implies that context can increase or decrease the motivation (21). Also, male students like to promote their social position while female students like to promote their learning skills (22).

Motivation is recognized as an important factor in health science education because it helps the students achieve good academic performance, well-being and satisfaction, and also helps them to become good professionals. However, research that centers on motivation in the health science disciplines is scarce. In the health professions, the long-term goal of professional career practice is an important component of motivation, which is related more closely with future competencies to be used in regulated professional practice than with knowledge and skills to be acquired in the learning process during university study (23). William showed that self efficacy construct can affect the intent, induce motivation to change, and contribute to practicing the following changes in motivation in medical students (24).

The study by Campos-Sanchez investigated the influence of different components of motivation (intrinsic motivation, self-determination, self-efficacy and extrinsic-career and grade-motivation) on learning human histology in health science curricula and their relationship with the final performance of the students in histology. Results showed that the overall motivational profile for learning histology differs among medical, dentistry and pharmacy students. This finding is potentially useful to foster their learning process, because if students are metacognitively aware of their motivation they will be better equipped to self-regulate their science-learning behavior in histology. This information could be useful for instructors and education policy makers to enhance curricula not only on the cognitive component of learning but also to integrate students' levels and types of motivation into the processes of planning, delivery and evaluation of medical education (23). Along with its varied influences on cognition, emotion also impacts motivation. For instance, positive emotions, such as the enjoyment and pride a clinician feels after helping a sick patient, may lead to more intrinsic motivation for the practice of medicine and its patient care-related aspects. Likewise, negative emotions, such as the frustration that can be felt when dealing with a difficult patient may decrease intrinsic motivation (25). Faculty development has been effective in improving faculty perceptions on the value of teaching, increasing motivation and enthusiasm for teaching, increasing knowledge and behaviors, and disseminating skills. So, faculty status can change the students’ motivation, too (26).

Since there are some concerns about motivational level in SUMS students, most faculty members in the talented students center believe that motivation decreases during education from the first entry level to the last year of education. Also, as evidence shows, there are many implicit and explicit factors that can affect the students' motivation. Therefore, the center for talented students aimed to design the present study to measure the achievement motivation level in all schools of the university and survey the factors that can affect it. 

## Methods

This study is a cross-sectional research conducted on undergraduate students (B.S. and M.D.) of ten schools in SUMS (Shiraz, southern Iran) in order to investigate their motivational level to study in their scientific field. The students were from different schools with various fields of study. They had entered university trough a national entrance exam held all over the country (Iran). Therefore, their selection was based on their scores in the entrance exam. Thirty percent of the students in SUMS were selected randomly by multi-stage stratified random sampling method. The research team selected the students randomly by systematic method from each science field and each entrance year in schools. Accordingly, 770 students entered the study. 

Based on the research objectives, two questionnaires were used in this survey. The first questionnaire was constructed by Hashemi in 1999 in Lamerd Azad University. Its validity and reliability have been confirmed in Iranian student population (%95 Cronbach's Alpha) (4). However, this coefficient was 85% in our study (statistically significant, p<0.001). It contains 57 questions to investigate the effect of economic, social, educational, geographical and personality factors on the students’ achievement motivation. To answer each question, students can choose one from five options, namely very high, high, medium, low, and very low indicating the effect of that factor on their academic motivation. Each question is analyzed separately in this questionnaire. The second one was achievement motivation scale (2). This questionnaire is based on 50 incomplete sentences. The students should complete them by one of three choices. For grading, the answers are compared to the standard answering key and the correct and wrong answers are assigned grade 1 and 0, respectively.  The total numbers give the final number for each student. Its validity was approved by the experts after its translation into Persian language and its reliability was calculated 90% using Cronbach's Alpha which is statistically significant (α-Coronbach= 0.9, p<0.001). Table 1 shows the standard categorization of the amount of students’ motivation according to their gender.

The sample students were asked to fill out these questionnaires accompanied by some biographic information. Their answers were then analyzed in SPSS for windows, version 16.0 (Chicago, IL) and Excel software. Some descriptive statistics are derived from the answers. And the results were compared in two genders, ten fields of study and among educational year using statistical tests such as Independent two samples t-test, ANOVA and Chi-square test (0.05 was considered as the significance level for statistical tests). 

## Results

For the first questionnaire, the answers include five ordinal options which measure the effect of these factors on academic motivation. To detect the importance of each factor on the SUMS' student motivation, the percentage of the students who selected the option "High" or "Very high" in each question was considered as the importance index of that factor. Table 1 summarizes the results on all the students. The first six factors which had the most effect on academic motivation were respectively chosen by the students as follows: "family attitudes towards education"," hope to get good jobs in future", "respect for themselves", "the ability to learn", "believe in his/her role in victory and defeat", and "the tendency toward optimism about themselves".  More than 80 percent of the students considered the effect of these factors important on their academic motivation. The last five factors with the least importance on academic motivation were "belief in chance and others' role in victory or defeat"," misery", " unemployment", " working while studying" and " student loans". These factors were selected by less than 40 percent of students. Accordingly, the importance of these 57 factors in academic motivation is statistically significant according to the students (Chi-square= 170.6, p<0.001).

**Table 1:** Percentage of students who chose 'High' or 'Very high' options for the effect of these factors on their academic motivation

**Factor**	**%**	**Factor**	**%**
Family attitude towards education	82.2	Use of educational aids	63.2
Hoping to get good job in future	81.8	Appropriate facilities in residence	61.9
Respect for themselves	81.6	Financial independence	61.1
Believing his/her role in victory and defeat	80.9	Method of examination	60.3
The ability to learn	80.9	Medical facilities	58.7
The tendency to optimism about themselves	80.5	Number of students in the class	56.2
Positive self image	79.3	Light, colours and feature of class	56.1
Commitment to the task	78.1	Employment in the public sector	55.9
Belief in the existence of opportunities for growth	77.7	Academic camps	55.4
Belief of their influence on events	77.5	Instructor skills	54.7
Successful models of education	76.3	Cultural centers at hand	54.2
To develop and compete with others	76.1	Green space of university	53.4
The importance of education in society	76.1	Private car	50.8
Social behavior of instructor	75.2	Regional climate	50.6
Social status of the family	73.4	Recreational camps	48.2
Educational facilities	73.1	Sport facilities at the university	47.7
Applied learning	72.5	Employment in the private sector	47.3
Family education	71.3	Anxiety	47.2
Academic atmosphere in universities	70.9	University counseling centers	45.4
Being in the academic environment	70.2	Increase in students' number	44.6
Known educational resources	70.0	Stay away from family	42.6
Friends' attitudes towards education	69.4	Celebrations and festivals	41.5
Financial support of parents	68.5	Lack of interest in participation with others	41.4
Living with family	67.7	Student loans	39.6
Teaching method	67.6	Working while studying	39.1
Professional floor	67.4	Unemployment	38.7
Appropriate educational space	65.8	Misery	34.8
Independent room	64.2	Belief in chance & others' role in victoryor defeat	32.1
Enjoy the team work and partnership	63.2		

The second questionnaire which is based on 50 incomplete sentences is a standard questionnaire used over the world to measure the academic motivation among the students. Its result was a score for each student which was calculated by comparing the answers to the standard question key. The total score of all questions was the final score for each person. It ranged from 0 to 44 for the students in our study. Table 2 shows the results of this questionnaire. Also, you can see the standard scoring of the questionnaire in Table 2.

**Table 2:** Categorization of study’s motivation for the second questionnaire

**Category**	**Grade**	
	**Boys**	**Girls**
High	23 and upper	23 and upper
Higher than medium	19-22	20-22
Medium	17-18	17-19
Lower than medium	14-15	14-16
Low	11-14	11-13

As a result, the total mean score was low compared to its maximum value (44 in our sample and 50 in standard one). In addition, no factor such as gender (T=-1.5, p=0.137), marital status (T=0.65, p=0.515), field of study (F=0.889, p=0.839), educational year (F=0.819, p=0.397), and their high school final score (F=0.935, p=0.423) had a significant effect on motivation score in SUMS students. The only significant factor was the special seat that the government has considered for university entrance of the students from the family of war martyrs and those from deprived areas of the country (F=3.942, p=0.020). 

Therefore, students who have no special seat have more motivation score than those from war martyrs’ family significantly. Furthermore, the comparison of two general fields of study (professional doctorate and basic sciences) showed that there was no significant difference between the mean score of the first and last year students (T=0.283, p=0.777). However, comparison with more details in eight fields showed different results. Dentistry and HMMI students revealed a significant decrease in motivation score during their education period according to the results of this study (F= 3.991, p=0.015). Table 3 summarizes the results.

**Table 3:** Analysis of mean and standard deviation of motivation score derived from the second questionnaire

**Factors**	**No. ** **(Valid Number)**	**Score** ** (Mean±SD)**	**p**
Total	766	19.79±5.63	-
**Gender **			
Man	214	19.31±5.37	0.137^a^
Woman	545	19.99±5.74	
**Marital status **			
Single	661	19.86±5.70	0.515
Married	96	19.46±5.37	
**High school final score **			
Less than 14	38	18.45±5.92	0.423^b^
14-17	384	19.74±5.69	
17-19	228	20.05±5.67	
19-20	106	20.02±5.40	
**Special seat for university entrance**			
Students who have no special seat	235	20.71±5.65	0.020^b*^
Students who have deprived area capacity	476	19.49±5.48	
Students from war martyrdom family	45	19.30±6.28	
**Field of study (occupation)**			
Professional doctorate (Medical & Dental)	274	19.85±5.32	0.839^a^
Basic sciences	492	19.76±5.80	
**Educational year **			
First	280	19.83±5.49	0.397^b^
Second	225	19.47±6.01	
Third	163	20.02±5.05	
Forth and higher (Last)	102	21.28±5.69	
**Changes between the first and the last years in eight fields**			
Medicine	123	-1.0±0.83^d^	0.777^c^
Dentistry	35	3.95±1.7	
Nursing and Midwifery	86	0.14±1.19	
Rehabilitation	47	0.58±1.55	
Paramedics	46	0.92±1.79	
Health and Nutrition	86	0.67±1.16	
Hospital Management and Medical Information (HMMI)	48	-2.6±1.83	
Nursing school in Lar (a small city in Fars Province)	45	1.02±1.58	0.015^b*^

^a^ Independent two- sample T-test

^b^ One way ANOVA test

^c^ Independent two- sample T-test (Comparison between First and Last educational year in the whole sample)

^d^ Mean and Standard Deviation of differences (First-Last)

^*^Significant at 0.05 level

Different category to the others by Tukey test

## Discussion

The present study was conducted to determine the achievement motivation level of SUMS's students during their studies in the university. Accordingly, a random sample of 770 students from 8 different fields of study participated in this research. Two standard validated questionnaires (one internal and the other international) were used for this purpose. The results showed that achievement motivation scores from “Acheivement Motivation Scale” that are related to personal factors and self efficacy were higher than the average (>19) in these students and it is a valuable finding for university policy makers to use.

Also, the six factors in questionnaire 1 as selected by 80% of the students were determined as the most effective factors on the level of the students' motivation. These 6 factors were related to personal and social groups. It is concluded that three important factors must be considered by university: 1) social, 2) personal, and 3) educational as other factors such as environmental, geographical and economic issues were of lower importance. As Boundra (1) argued, behavior was influenced by a combination of social and personal characteristics such as beliefs about possible outcomes of behavior and their ability to promote a given task competency. Personal attitude and self-efficacy can be induced by modeling, previous successful experiences, and social comparison information.

Therefore, the combination of social and personal factors was important to increase the students’ motivation for learning and hard working (4). In a qualitative research, Mariette Bengtsson found that motivation must come from the students themselves, but dedicated teachers who give feedback on the performance of their students and discuss about  different forms and choices of learning and assessment methods can enhance enthusiasm and learning in their students (18).

By providing an educational environment that matches with the students’ needs, teachers might be successful in their teaching. Nowadays, students are a heterogeneous group of adults with different ages, backgrounds, and social positions, and their ability to cope with the situation of being a university student differs (19). The results of this research indicated that environmental factors cannot affect the level of motivation and it is a doubtful finding and needs more research in the future to be confirmed.

As the results showed, there were no significant differences in achievement motivation scores between male and female, married and single, professional doctorate (Medical & Dental) and basic sciences, and first and last years of education. So this demographical condition could not affect achievement motivation. As mentioned, there was no significant difference between male and female participants in achievement motivation. Gang confirmed it in previous research. On the contrary, Darabi found that motivation is different between male and female students (11, 13). In addition, Yousefi found that achievement motivation is related to the students’ mean scores in each term. But, he did not consider the effect of high school scores on motivation in the university. However, the present research showed no significant impact of high school final scores on the students' motivation (F=0.935, p=0.423).

Furthermore, some evidence on the comparison of motivation between the first and last educational years students (junior and senior) showed that academic motivation improved only for 34% of them during the first 4 years of education and the others remained fixed (19). Also, in this study, the results indicated no significant differences in achievement motivation between the first and last year of entry. Consequently, the hypothesis of decrease in the academic motivation in Iranian students with increase in educational year is not generally confirmed by our survey. However, dentistry and HMMI students showed a significant decrease in motivation score during their educational years (F=3.991, p=0.015), the point which requires further attempts to study in detail. The results of this research may be useful for SUMS's authorities in charge of education, research and welfare departments. Therefore, better planning can be done in providing the students with necessary facilities in order to improve their motivation in their education period and future job. In addition, the management of university entrance exams all over the country should improve the exam and consider the students' interests in selecting their field of study.

## Conclusion

The findings of this study support those of the previous studies. For instance, intrinsic factors can improve the students’ attitudes more than extrinsic ones. So, authorities have to consider our findings in devising university entrance exam. However, if we fit the students with the field of the study and study major, we will gain more optimal educational results. Furthermore, since motivation scores were derived from the questionnaires in this study, the time in which the students were asked to answer the questions is very important. Feeling comfortable in non-stressful environments affects the answers and this can be mentioned as the limitation in this research. 
